# Modeling Neurovascular Coupling from Clustered Parameter Sets for Multimodal EEG-NIRS

**DOI:** 10.1155/2015/830849

**Published:** 2015-05-19

**Authors:** M. Tanveer Talukdar, H. Robert Frost, Solomon G. Diamond

**Affiliations:** ^1^Thayer School of Engineering at Dartmouth, 14 Engineering Drive, Hanover, NH 03755, USA; ^2^Institute for Quantitative Biomedical Sciences, One Medical Center Drive, Lebanon, NH 03756, USA

## Abstract

Despite significant improvements in neuroimaging technologies and analysis methods, the fundamental relationship between local changes in cerebral hemodynamics and the underlying neural activity remains largely unknown. In this study, a data driven approach is proposed for modeling this neurovascular coupling relationship from simultaneously acquired electroencephalographic (EEG) and near-infrared spectroscopic (NIRS) data. The approach uses gamma transfer functions to map EEG spectral envelopes that reflect time-varying power variations in neural rhythms to hemodynamics measured with NIRS during median nerve stimulation. The approach is evaluated first with simulated EEG-NIRS data and then by applying the method to experimental EEG-NIRS data measured from 3 human subjects. Results from the experimental data indicate that the neurovascular coupling relationship can be modeled using multiple sets of gamma transfer functions. By applying cluster analysis, statistically significant parameter sets were found to predict NIRS hemodynamics from EEG spectral envelopes. All subjects were found to have significant clustered parameters (*P* < 0.05) for EEG-NIRS data fitted using gamma transfer functions. These results suggest that the use of gamma transfer functions followed by cluster analysis of the resulting parameter sets may provide insights into neurovascular coupling in human neuroimaging data.

## 1. Introduction

Neural activity is a complex biophysical process that involves electrochemical and vascular interaction at the cellular level. Modern neuroimaging technologies such as functional magnetic resonance imaging (fMRI), near infrared spectroscopy (NIRS), and diffuse optical imaging (DOI) typically detect changes in the local blood flow associated with the vascular response to provide an indirect measure of neural activity. A major limitation with these techniques is their inability to map precisely the source of the underlying neural activity [1, 2]. Consequently, understanding neurovascular coupling or the relationship linking the underlying neural activity to the local changes in cerebral hemodynamics remains a vital area of research.

To date, noninvasive multimodal neuroimaging techniques with electroencephalography (EEG) and fMRI have been widely used to investigate the relationship between neural activity and cerebral hemodynamics [3–5]. These studies attempt to show quantitative aspects such as the degree of correlation between electrical scalp potentials recorded using EEG and the hemodynamic response measured from fMRI after separately preprocessing the acquired data. Only a few studies have investigated the direct link between neural activity and the hemodynamic response by estimating transfer functions which map neural rhythms recorded using EEG to fMRI blood-oxygen level dependent (BOLD) signals reflecting the hemodynamic response. Despite being informative, there are a number of methodological and technical difficulties associated with multimodal EEG-fMRI techniques. EEG data recorded during fMRI acquisitions can be contaminated by gradient artifacts induced by the changing magnetic field gradients used for spatial encoding in MRI [6]. Electromagnetic interference could decrease the signal quality in both modalities and therefore obscure features of interest in the datasets being used to identify the neurovascular coupling relationship. Moreover, EEG-fMRI instrumentation can be fairly complex to set up and is not readily accessible for conducting neuroimaging research in smaller research facilities.

Multimodal neuroimaging with EEG and NIRS is increasingly gaining popularity due to the portability of EEG-NIRS instrumentation. EEG-NIRS systems can provide measurements with high signal-to-noise ratio (SNR) since there is no electrooptical interference. More importantly, NIRS can measure the hemodynamic changes at the capillary level while fMRI detects BOLD signal that is sensitive to changes in venous outflow [7]. This makes NIRS potentially advantageous for measuring hemodynamics associated with neural activity as there is evidence which suggests that neuronal function is supported by brain capillary oxygenation [8]. A number of studies have used EEG-NIRS systems to investigate neurovascular coupling from the measurement of the electrical scalp potential and the vascular response in stimuli evoked neural responses [9–11]. Although these prior works show that neural correlates of EEG signals covary with NIRS hemodynamics, there is still not a clear understanding of how the signals measured with EEG are predictive of NIRS. Newer methods need to be established based on models that relate neural activity to the hemodynamic response.

In this work we introduce a neurovascular-coupling modeling approach based on fitting gamma transfer functions and then clustering the parameters into sets that most effectively map the electrical scalp potentials recorded using EEG to the cerebrovascular response measured from NIRS hemodynamics. Gamma transfer functions have been used to represent the hemodynamic response function (HRF) in fMRI analysis methods [12–14]. Typically in fMRI data analysis, the BOLD signal is regressed on a general linear model (GLM) constructed by convolving the HRF with a boxcar function representing the temporal structure of the experimental paradigm [15]. In recent years, NIRS studies have also adopted the GLM framework to map brain activations [16]. Unfortunately, a boxcar function convolved with a canonical HRF may not accurately represent the cerebrovascular response since there is variability in the neural response between brain regions and different tasks [17, 18]. In addition, the shape of the HRF will depend on the type of imaging modality used [19]. NIRS hemodynamics, which have higher temporal resolution than fMRI BOLD signals and different hemodynamic measures, will require a different parameter set for the HRF compared to parameters used in the BOLD fMRI. With respect to the GLM modeling framework, our neurovascular-coupling modeling approach takes advantage of the measured EEG signal linked directly to neural rhythms in order to predict the NIRS hemodynamic response. Moreover, we vary the parameters of the gamma transfer function so that our model predictions have improved fit. Finally, using our approach we want to identify reliable gamma transfer function parameters that can predict NIRS ΔHbO hemodynamic response from EEG spectral envelopes within an individual.

## 2. Methods

We conducted an Institutional Review Board (IRB) approved experiment that involved unilateral stimulation of the median nerve. Typically, stimulation of the median nerve on the hand induces mu rhythm desynchronization in the 8–13 Hz range in the contralateral somatosensory area of the brain. The cortical response can be detected as fluctuations in the EEG mu band and is also known to produce positive changes in ΔHbO calculated from NIRS optical measurements [20, 21]. Our experimental paradigm is designed in order to facilitate detection of signal components that correlate with the median nerve stimulus. In this way we have selected for signals that are likely to have originated in the same brain region, which makes it practical to study neurovascular coupling.

In our study we use an EEG-NIRS head probe that was designed to record neural activity from optimally placed EEG electrodes and NIRS optodes, which cover the whole head [22]. The positioning of the EEG sensors is configured so that they are equidistant and close in proximity to a NIRS optode. This ensures to some degree that the measured signals from an EEG electrode and a NIRS optode have a common source in the cerebral cortex. The EEG-NIRS instrumentation consists of 16 optical sources and 8 detectors interleaved with EEG sensors, which are arranged according to the International 10–20 system. The NIRS system emits continuous wave (CW) near infrared light at four wavelengths (690 nm, 785 nm, 808 nm, and 830 nm). The source-detector spacing is 33 mm, which allows for probing neural activation with high sensitivity [23]. The EEG system measures the electrical scalp potentials from 30 electrodes. The data acquisition rates for the EEG and NIRS systems are 2048 Hz and 25 Hz, respectively.

### 2.1. Data Preprocessing

EEG data is contaminated by ocular, muscular, cardiac, and other physiological artifacts. For the sampled EEG data, epochs containing aberrant waveforms were manually discarded by visual inspection using the EEGLAB TOOLBOX 10.2.2.4b (Swartz Center for Computational Neurosciences, La Jolla, CA; http://www.sccn.ucsd.edu/eeglab/). We then applied independent component analysis (ICA) using the Infomax ICA algorithm [24], as implemented in EEGLAB to remove ocular movement and blink artifacts from the EEG data [25–27]. Empirical mode decomposition (EMD) was next applied to the motion corrected and noise artifact reduced EEG data for extracting neural rhythms linked to mu rhythm desynchronization. EMD decomposes a signal into a set of band limited functions called intrinsic oscillatory modes or integral mode functions (IMF) [28]. Each IMF represents oscillations in a narrow frequency band and may reveal particular features of interest in the given signal. The EMD method can offer better time-frequency localization compared to traditional methods like short time Fourier transform (STFT) and wavelet decomposition techniques [29, 30]. STFT has fixed time-frequency resolution due to the fact that it gives a global frequency distribution in the processing time window. Wavelet analysis, on the other hand, can provide variable time-frequency resolution compared to STFT method. However, there is a trade off between the temporal and spatial scales depending on choice of the wavelet basis function. In EMD, one does not require any basis functions and the signals do not need to be stationary, as is assumed in STFT and wavelet analysis methods. As a result EMD has gained considerable popularity for the analysis of EEG data, which exhibit a high degree of nonlinearity and oscillatory rhythms in subband frequencies [31, 32].

NIRS optical signal can be contaminated by motion artifacts and sources of physiological noise such as cardiac signals. Motion artifacts were rejected by applying Chauvenet's criterion [33]. A deviation ratio (DR) at each time point was calculated by dividing the NIRS signal deviations by the standard deviation of the signal deviations. Signal deviations were calculated as the difference between the raw data and its smoothed version obtained by applying a moving average filter with a span of 30 points (1.2 s duration). Data with a DR greater than the standard Chauvenet's criterion threshold were eliminated and the discontinuous data segments then spliced together. The resulting NIRS signal was then used to calculate changes in hemodynamics by solving equations of the modified Beer-Lambert law (MBLL), which relates the attenuation of light to relative changes in concentration of oxy-hemoglobin (ΔHbO) and deoxy-hemoglobin (ΔHbR) [34]. ΔHbO and ΔHbR were then detrended and low pass filtered using a zero-phase 3rd order Butterworth filter with a cutoff frequency of 0.5 Hz to remove cardiac oscillations, which contaminate NIRS signal [35].

**Figure 1:**
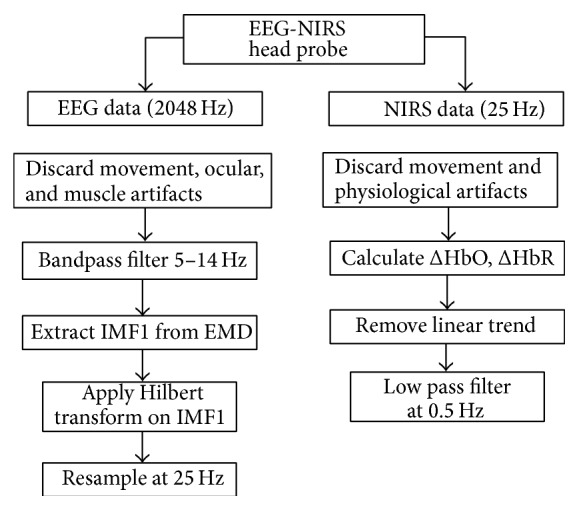
Flowchart: EEG-NIRS data preprocessing.

Figure 1 shows a flowchart of the preprocessing stream for the simultaneously acquired EEG-NIRS data during median nerve stimulation. EEG and NIRS data were sampled at 2048 Hz and 25 Hz, respectively, using the prototype EEG-NIRS head probe. Both EEG and NIRS data were treated for artifact rejection and were matched temporally across sampled data points. The EEG recordings were bandpass filtered (5–14 Hz) using a zero-phase 4th order Butterworth filter in order to contain the mu rhythm band (8–13 Hz). Hilbert transform [36] was then applied on the first IMF signal component (IMF1) to generate EEG spectral envelopes, which was later downsampled to 25 Hz. The EEG spectral envelope corresponding to IMF1 was chosen because it is modulated by frequency components in the mu band and is temporally correlated to the NIRS hemodynamic response due to the median nerve stimuli. The EEG spectral envelope and NIRS hemodynamics also have similar timing and bandwidth in the frequency domain.

### 2.2. Neurovascular-Coupling Model

In our model of neurovascular coupling, we assume that gamma transfer functions can map EEG spectral envelopes containing neural rhythms to NIRS ΔHbO reflecting the hemodynamic response linked to the underlying neural activity. This mapping can be expressed by

(1) $$\begin{array}{c} f\left(t\right)=a\left(g\left(t\right)*h\left(t;\tau ,n,d\right)\right)+b, \end{array}$$

where *g*(*t*) is an EEG spectral envelope, which is convolved with a candidate gamma transfer function *h*(*t*; *τ*, *n*, *d*) to predict the hemodynamic response *f*(*t*). The variables *a* and *b* are the gain and offset, respectively. Gamma transfer functions can be expressed in exponential form with parameters *τ*, *n*, and *d* [37]:

(2) $$\begin{array}{c} h\left(t;\tau ,n,d\right)=\frac{{\left[\left(t-d\right)/\tau \right]}^{n-1}e^{-(t-d)/\tau }}{\tau \left(n-1\right)!}, \end{array}$$

where *τ* determines the rise time to peak amplitude, *n* is an integer which governs the shape of the function, and *d* is a pure delay. In our method, we recover the parameters *τ*, *n*, and *d* of the gamma transfer function that produces the best fit between the observed and predicted NIRS hemodynamics. Identification of the gamma transfer function and its parameters are carried out in two steps. In the first step, a brute force technique is applied to calculate values *a* and *b* in (1) by least squares inversion for each gamma transfer function constructed from a set of predefined parameters *τ*, *n*, and *d*. These predefined parameter values (*τ* = 0.1–0.6 s, *n* = 1–4, and *d* = 0.5–3 s) were empirically chosen based on the observation of NIRS ΔHbO response time to median nerve stimulation. Usually the delay in ΔHbO response time can vary between 2 and 3 s, while the time to peak can be fairly quick 0.1–0.5 s [38]. From the solution space, we then identify *a*, *b*, *τ*, *n*, and *d*, which minimize the residual sum squared error (SSE). In the second step, the identified gamma function parameters (*τ*, *n*, and *d*) including the gain *a* and offset *b* are jointly optimized using a simplex search method [39]. This is performed using Maltab's “fminsearch” routine in which we specify SSE as the objective function.

### 2.3. Estimating Gamma Transfer Functions for Simulated EEG-NIRS Data

We simulated EEG data reflecting mu rhythm desynchronization associated with neural responses in the somatosensory cortex [40]. The artificially produced EEG data was in the form of an amplitude modulated sinusoidal signal having a carrier frequency of 9 Hz. A rectangular pulse train with eight pulses having a width and period of 20 s and 40 s, respectively, was used to modulate the sinusoidal signal. This pulse train was first convolved with a Kaiser window having a size of 250 data points. This smoothed the edges of the pulse train and introduced a time shift of approximately 5 s in the pulse onset times. The sign of the time shifted pulse train was then reversed to represent the spectral envelope for mu rhythm desynchronization events that diminish in power during the stimulus. This was then used to generate the simulated EEG signal by standard amplitude modulation operation given by

(3) $$\begin{array}{c} y\left(t\right)=\left[1+m\left(t\right)\right]\cdot c\left(t\right), \end{array}$$

where *m*(*t*) is the modulation wave, *c*(*t*) is the carrier wave, and *y*(*t*) is the modulated signal. The EEG signal thus contained eight 20 s mu rhythm desynchronization events with a latency of 5 s at each onset time.

NIRS ΔHbO was generated from the forward model of (1). Gamma transfer functions were evaluated for five different combinations of *τ*, *n*, and *d* that ranged in values from 0.3–0.6 s, 2–4, and 2-3 s, respectively. The gain *a* and offset *b* were set to 1 and 0. Each of the 5 gamma transfer functions was next convolved with the amplitude signal of the first IMF (IMF1) obtained from decomposition of the simulated EEG by the EMD technique. IMF1 basically had a spectral peak around the carrier frequency of 9 Hz and its amplitude signal was calculated from the Hilbert transform [36]. Both the lMF signal and NIRS ΔHbO were normalized in their amplitudes by dividing by their respective standard deviations. We then titrated both the EEG IMF1 signal and NIRS ΔHbO with varying degrees of white Gaussian noise (WGN) at each epoch. In case of ΔHbO, we also added a sinusoidal signal component of 0.5 Hz frequency modulating the WGN. The resulting ΔHbO were representative of more realistic NIRS measurements, which contain physiological noise such as cardiac oscillations that are in the 0.4–2.0 Hz range [41]. The amplitude ratio (AR) of the WGN was selected to be in the range from −*∞* dB (no noise) to 16 dB.

After generating the 5 EEG-NIRS datasets, we estimated the known gamma transfer functions that were used to model the NIRS data and recover the corresponding parameters *τ*, *n*, and *d*. We applied the brute force and the simplex search method to calculate the gamma transfer function parameters for each EEG-NIRS epoch data segments that spanned the pulse start times to 15 s past their end times. The parameters *τ*, *n*, and *d* identified at each epoch data segments were then used in the forward model of (1) to predict the simulated ΔHbO. The Pearson's correlation between the simulated and predicted ΔHbO was then evaluated at each amplitude ratio of the WGN.

### 2.4. Estimating Gamma Transfer Functions for Experimental EEG-NIRS Data

Gamma transfer functions that provide insight into the neurovascular coupling relationship in humans were estimated from EEG-NIRS data measured from three right handed male subjects (mean age of 31) in an IRB approved study. The subjects participating in the study had no history of neurological or psychiatric disorders and were in general good health. The left and right median nerves were stimulated using an electrical pulse generator that operated at 10 Hz. Pulses were transmitted during a 15-second block interval followed by 30 second rest interval during each experimental run that lasted a total of 6 minutes. Five recording sessions were carried out, two for each of the left and right median nerve stimulation runs and and one for which the subject was at rest and no stimulation was applied.

IMF1 spectral envelopes were derived for each of the 30 EEG channels. We then selected sets of IMF1 spectral envelopes corresponding to EEG channels located in the left and right hemispheres based on how strongly they correlated with the electrical pulse sequence. Similarly, NIRS ΔHbO signals were selected within each hemisphere if they showed changes in concentrations above 0.5 *μ*M. For each EEG IMF1 spectral envelope and NIRS ΔHbO paired dataset within a hemisphere, we extracted epochs spanning the stimulus interval of 15 s including 5 s of pre- and poststimulus period. The epoch data were later smoothed with a moving average window having a time span of 4 s and normalized by dividing by their standard deviation. Each of the epoch datasets consisting of normalized IMF1 spectral envelope and NIRS ΔHbO finally served as the neurovascular-coupling model input and output, respectively. Gamma transfer functions were then estimated for each epoch data segment using the two-step method described in Section 2.2.

We next investigated whether the estimated gamma transfer functions could be clustered into groups which are statistically significant in their predictions of the NIRS ΔHbO hemodynamics. By clustering the estimated gamma transfer functions, we hope to reduce the effect of overfitting in our neurovascular-coupling model. In addition, the clustering method is intended to identify reliable gamma transfer functions which link the EEG input to the NIRS hemodynamic output at the individual level. For each of the estimated gamma transfer functions, the parameters *τ*, *n*, *d*, gain *a*, and offset *b* were used to form a 5-dimensional feature vector or data point. Since the features were scaled differently, we standardized the feature space so that they have zero mean unit variance. Hierarchical clustering was then applied on the feature space derived from all the standardized parameter feature vectors comprised of *a*, *b*, *τ*, *n*, and *d*. We chose this clustering method due to its relative ease of implementation and also because it is widely used as a clustering tool. In hierarchical clustering, data is grouped by linking feature vectors in a binary tree called a dendrogram [42]. There are a number of linkage methods to merge the feature vectors into clustered parameter sets. We used Ward's linkage method as it has been shown to have better clustering performance [43]. In this method the within-cluster variance is minimized over all partitions obtainable by merging two clusters from the previous generation.

By specifying threshold values for cutting off the dendrogram at specific depths, we clustered the feature vectors into cluster divisions *C*_*N*_, where *N* = 2,3,…, 12. For each clustered parameter set, we calculated the Pearson's correlation coefficient *r* between the predicted NIRS ΔHbO obtained using the forward model (Equation (1)) and the measured ΔHbO. The correlations were Fisher *Z* transformed to give *z* values that are approximately standard normal in their distribution. The mean *z* value, $\bar{z}$, was then computed for each of the clustered parameter sets. The $\bar{z}$ value was next tested for statistical significance by performing a two-sided *Z*-test under the null hypothesis *H*_0_ that $\bar{z}$ = 0, which corresponds to a correlation coefficient of zero. This was done by first computing the *Z* score, *z* ^*^, from the relationship:

(4) $$\begin{array}{c} z^{*}=\sqrt{n}\bar{z}, \end{array}$$

where *n* is the number of feature vectors in the cluster. This *z* ^*^ value was then compared against the standard *Z* score *z*_*α*_ at the 5% significance level (*α* = 0.05). It is important to note that the *P* values generated via this two-sided *Z*-test are only approximate, are likely biased towards more extreme values, and should not be interpreted as accurate measures of statistical significance. This inaccuracy is due to both the fact that the underlying EEG-NIRS data does not follow independent observations from a bivariate normal distribution under the null hypothesis, giving the Fisher-transformed Pearson correlation coefficients a distribution different from *N*(0,1), and the fact that *Z*-statistics computed for each gamma transfer function parameter vector within a given cluster are not independent, increasing the variance of the mean of the *Z*-statistics. Despite this inaccuracy, the computed *P* values can still be successfully used to help to select the optimal split level for the dendrogram and to identify clusters of gamma transfer function parameter vectors that closely model the empirical EEG-NIRS relationship and merit more detailed analysis and interpretation.

Due to the fact that we are performing *N* significance tests on $\bar{z}$ values in *C*_*N*_ cluster divisions, the *z*_*α*_ values were adjusted for multiple hypothesis correction (MHC) using the Bonferroni correction [44]. For each cluster division 2 to 12, we also recorded the number of significant clusters by counting *z* ^*^ values that exceeded *z*_*α*_. From this count we assessed the optimal *C*_*N*_ that the data can be divided into using hierarchical clustering. Our approach to MHC was to control the family-wise error rate (FWER) using the Bonferroni method for the family of hypothesis tests associated with the two-sided *z*-tests performed for each of the clusters generated by one split of the dendrogram. Although MHC for this problem could have been used to control the false discovery rate (FDR) rather than the FWER or been applied to the larger family of hypothesis tests associated with all dendrogram splits, we choose the current approach to facilitate selection of the optimal dendrogram split level.

## 3. Results and Discussion

### 3.1. Simulated EEG-NIRS Data

**Figure 2:**
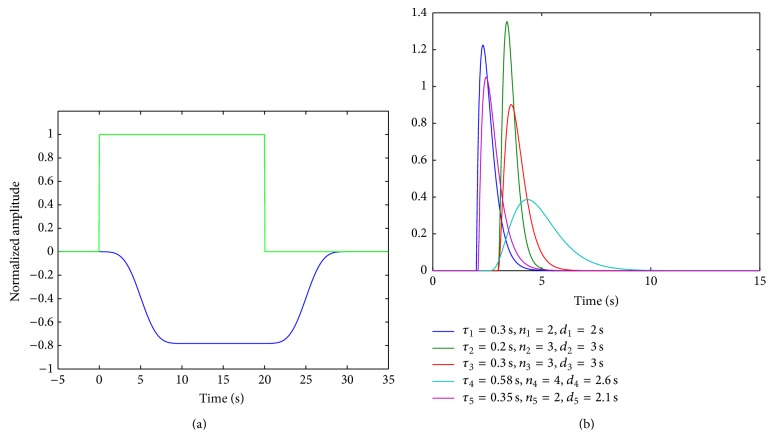
(a) Simulated EEG IMF1 envelope (blue) and pulse (green) and (b) Gamma transfer functions used to generate NIRS ΔHbO.

Figure 2(a) shows an epoch of the simulated IMF1 spectral envelope (blue) and the pulse duration (green). The 5 gamma transfer functions that were used to simulate NIRS ΔHbO are shown in Figure 2(b). The parameters of the gamma transfer function were chosen heuristically based on the notion that they reflect physiologically relevant NIRS hemodynamic response with respect to latency, shape, and peak time behavior.

**Figure 3:**
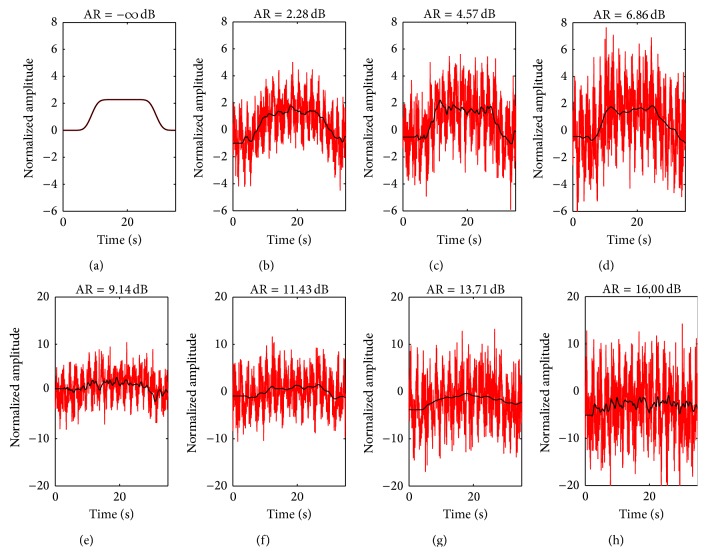
((a)–(h)) Simulated NIRS ΔHbO (red) at different noise amplitude ratios (AR) for WGN; estimated NIRS ΔHbO (black). Note that vertical scale of top row is different from bottom row.

Figures 3(a)–3(h) show simulated noise titrated ΔHbO epoch data (red) obtained from convolution of the IMF1 spectral envelope in Figure 2(a) with a gamma transfer function having parameters *τ* = 0.3 s, *n* = 2, and *d* = 2 s (see gamma function in blue, Figure 2(b)). For each of the 8 ΔHbO epoch data, the estimated ΔHbO are shown overlaid in black. Perfect fit is obtained when the noise AR is −*∞* dB, which is practically zero noise level. At higher levels of noise, the estimated ΔHbO has poorer fits with the simulated ΔHbO. However, the estimated ΔHbO in Figures 3(b)–3(d) seems to follow the dynamics of the noise-free simulated ΔHbO in Figure 3(a). At noise AR of 9 dB and higher, the estimated ΔHbO no longer exhibit the dynamics of noise-free simulated ΔHbO. Similar trends are observed for ΔHbO epoch data simulated using the other gamma transfer functions.

**Figure 4:**
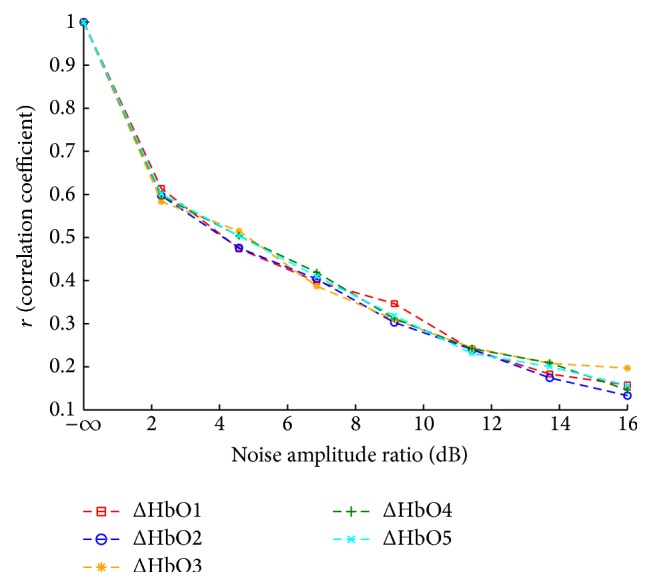
Correlation coefficient between estimated NIRS ΔHbO and simulated ΔHbO for different noise amplitude ratios in WGN.

Figure 4 shows the Pearson's correlation *r* values between the estimated ΔHbO and the simulated ΔHbO numbered 1 through 5 against noise AR in WGN. For the noise-free (AR = −*∞* dB) simulated ΔHbO numbered 1–5, *r* = 1, indicating that the estimated and simulated ΔHbO are in agreement with each other with a goodness of fit statistic *R* ^2^ = 1. There is a sharp drop in *r* value from 1 to 0.6 as AR increase from −*∞* dB to 2.2 dB. At higher noise amplitude ratio from 2.2 dB to 11.5 dB, *r* values drop almost linearly with a gentler slope. Between AR values of 12 dB to 16 dB, the *r* values can be seen to level off. From this analysis we can note that reliable estimates of NIRS ΔHbO, which is strongly correlated with the simulated ΔHbO (*r* > 0.5) can be obtained when the noise AR is below 4 dB.

**Figure 5:**
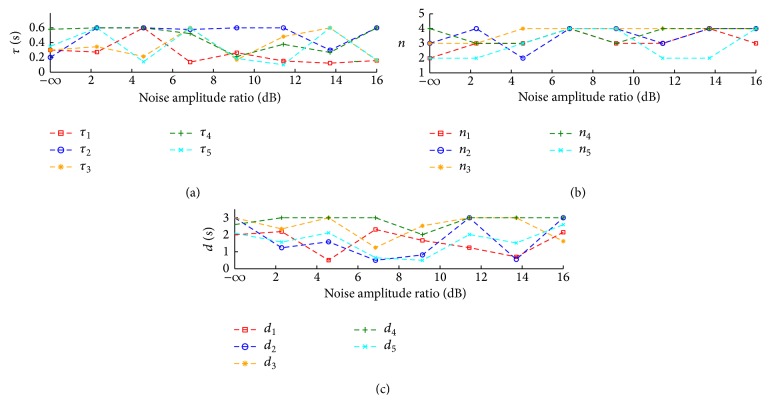
Recovered parameters of the fitted gamma transfer functions for the 5 simulated EEG-NIRS datasets at different noise amplitude ratios: (a) *τ* (peak time), (b) *n* (shape), and (c) *d* (pure delay).

Figure 5 shows parameters recovered at different AR values for the 5 sets of NIRS ΔHbO generated from the known gamma transfer functions. We can note that, for AR = −*∞* dB, the parameters recovered from the gamma transfer functions fits are identical to the original ones used to simulate ΔHbO (see Figure 2(b)). However, all the recovered gamma transfer function parameters vary from the original ones for subsequent fits in which the AR values of the WGN increase from 2.2 dB to 16 dB. However, a number of the estimated gamma transfer function parameters remain stable in their value up to certain noise AR ratio. For example, the recovered parameters *τ*, *n*, and *d* for noise AR of 2.2 dB to 6.86 dB agree closely with the known gamma transfer function parameters with *τ* = 0.58, *n* = 4, and *d* = 2.6 (green dotted line). A similar observation can be made for the gamma transfer function with parameters *τ* = 0.3, *n* = 3, and *d* = 3 (orange dotted line) for which the parameter estimates of *τ*, *n*, and *d* agree closely with the known values as the noise AR increases from −*∞* dB to 4.57 dB. It is also interesting to note that, in Figure 5(b), the shape parameter for 4 of the 5 gamma transfer functions seems to level off at around *n* = 4 for noise AR greater than 6.86 dB. Despite the differences between the recovered and known values of the gamma transfer function parameter at increasing noise AR, our neurovascular-coupling model is still able to predict the dynamics of the simulated NIRS ΔHbO containing moderate levels of WGN (see Figure 3).

### 3.2. Experimental EEG-NIRS Data

**Figure 6:**
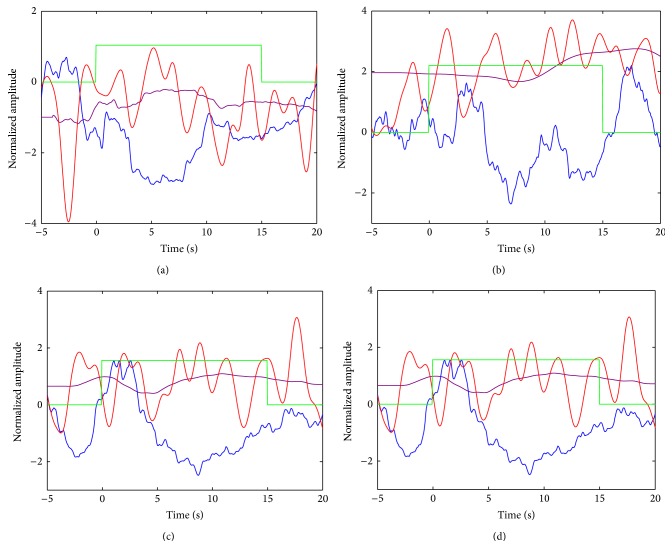
(a)–(d) show results from fitting gamma transfer functions to 4 EEG-NIRS datasets or epochs, each of which corresponds to a pulse period including 5 s of pre- and poststimulus interval. The EEG IMF1 envelope (blue), low pass filtered NIRS ΔHbO (red), the estimated NIRS ΔHbO (purple), and the pulse duration (green) are shown in each plot.

Figures 6(a)–6(d) show sample results from fitting experimental EEG-NIRS data from Subject 1. In each plot, the estimated NIRS ΔHbO (purple) is overlaid on the calculated ΔHbO (red). The EEG IMF1 spectral envelope is shown in blue and the pulse duration (15 s) in green. The EEG-NIRS data displayed in each plot have been normalized by their standard deviations. We can note that, after the onset of the stimulus at 0 s, the EEG IMF1 envelope decreases in amplitude while the NIRS ΔHbO show a steady increase.

**Figure 7:**
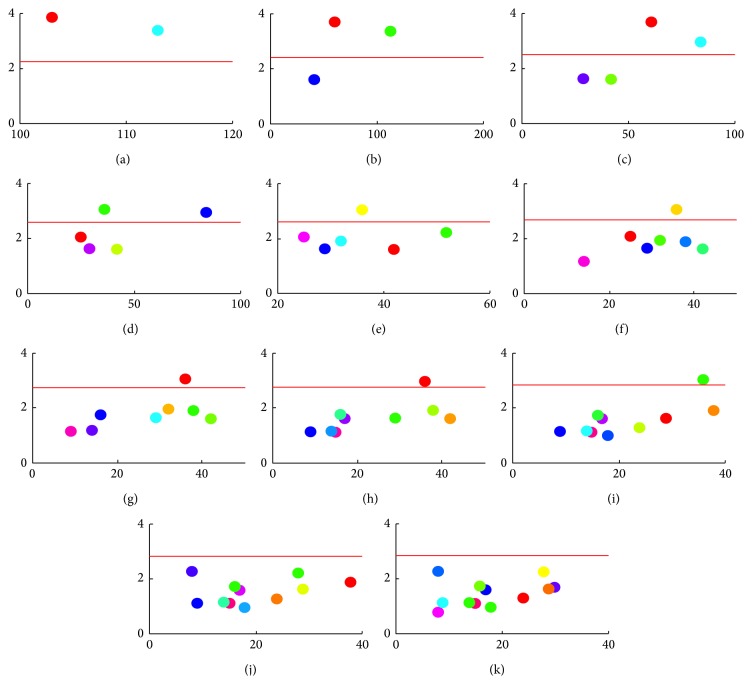
(a)–(k) Plot of cluster *z* ^*^ values (colored dots) against number of feature vectors (*x*-axis) for each cluster division *C*_*N*_ for subject 1. The statistical threshold for the two sided *Z*-test at the 5% significance level is indicated by the horizontal red line.

Figures 7(a)–7(k) shows cluster *z* ^*^ values computed for cluster division *C*_*N*_ ranging from 2 to 12 in subject 1's left hemisphere during right median nerve stimulation. The total number of feature vectors used in hierarchical clustering for this subject and experimental condition was 216. The statistical threshold *z*_*α*_ for the two sided *Z*-test, which was adjusted for multiple comparison by Bonferroni correction, is indicated by the red line in each plot. The number of significant clusters *C*_*S*_ with *z* ^*^ exceeding *z*_*α*_ decreases with an increasing number of cluster divisions. For cluster divisions 11 and 12, there are no significant cluster *z* ^*^ values.

**Figure 8:**
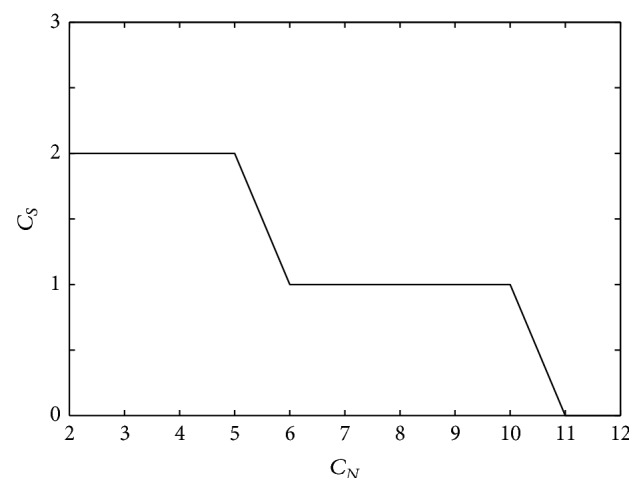
Number of cluster divisions (*C*_*N*_) versus number of significant clusters (*C*_*S*_) for subject 1.

Figure 8 shows the relationship between *C*_*S*_ and *C*_*N*_, which were obtained from the plots shown in Figure 7. Since cluster divisions 2 to 5 have the highest number of significant clusters (*C*_*S*_ = 2), we take the optimal *C*_*N*_ to be 5. This is based on the assumption that a higher number of clustered gamma transfer function parameters sets have greater flexibility for modeling the EEG-NIRS relationship compared to fewer clustered parameter sets.

**Figure 9:**
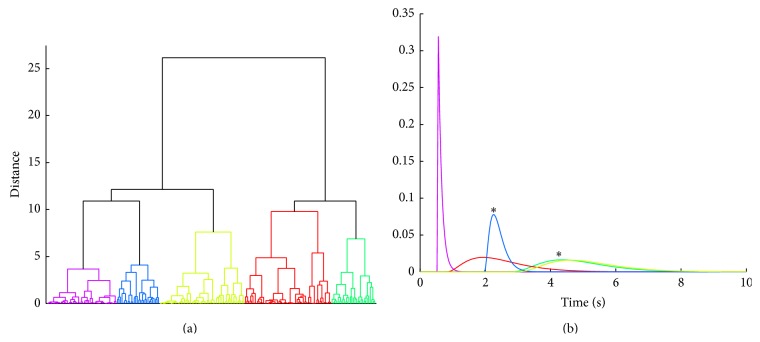
(a) Dendrogram for optimal *C*_*N*_ = 5 in subject 1. (b) Representative gamma transfer functions color matched to the clustered parameter sets in the dendrogram. The gamma transfer functions colored blue and green correspond to the statistically significant *z* ^*^ cluster values shown in Figure 7(d) in the same colors (∗indicates significance).

Figure 9(a) shows the dendrogram obtained at optimal cluster division *C*_*N*_ = 5 in subject 1 (Figure 8). The representative gamma transfer functions for the clustered parameters sets in the dendrogram are shown in Figure 9(b). Each of these functions was generated by taking the mean of the gamma transfer function parameters values corresponding to individual cluster groups. Their amplitudes were then scaled by dividing by the respective area under the curve. The blue and the green gamma transfer functions correspond to the cluster *z* ^*^ values that were statistically significant; that is, *z* ^*^ > *z*_*α*_. The gamma transfer function in blue has a delay of about 2 s and peak time that is less than 0.5 s. In contrast, the gamma transfer function in green has a larger delay of approximately 3 s and a longer peak time of about 1 s.

**Figure 10:**
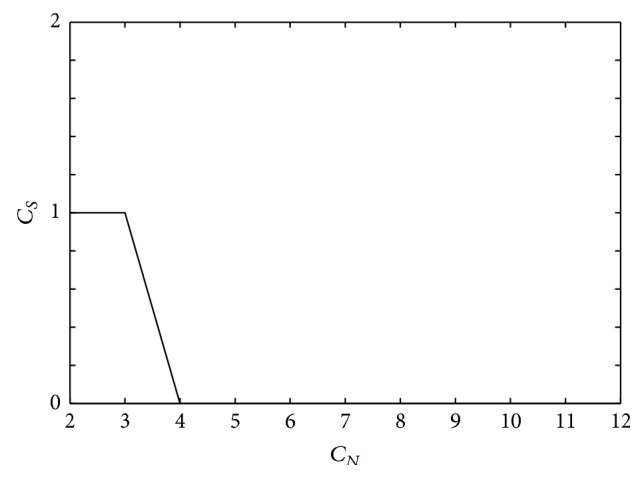
Number of cluster divisions (*C*_*N*_) versus number of significant clusters (*C*_*S*_) for subject 2.

Figure 10 shows subject 2 s statistically significant clusters *C*_*S*_ for each cluster division *C*_*N*_. Hierarchical clustering was performed on feature vectors obtained by fitting EEG IMF1 envelopes to NIRS Δ*HbO* sequences in the left hemisphere during right median nerve stimulation. There were 96 feature vectors for this subject and experimental condition. Based on the graph of *C*_*N*_ versus *C*_*S*_ in Figure 10, the optimal cluster division is *C*_*N*_ = 3 and the number of significant clusters is *C*_*S*_ = 1.

**Figure 11:**
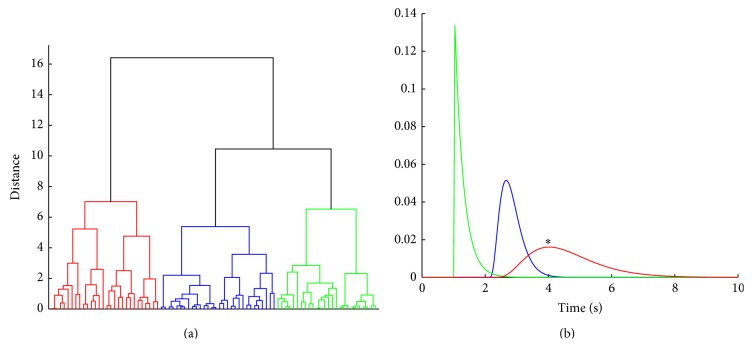
(a) Dendrogram for optimal *C*_*N*_ = 3 in subject 2. (b) Representative gamma transfer functions color matched to the clustered parameter sets in the dendrogram. The gamma transfer functions colored red correspond to the statistically significant *z* ^*^ cluster value (^*^indicates significance).

Figure 11(a) shows subject 2 s dendrogram for optimal cluster division *C*_*N*_ = 3 identified in Figure 10. The representative gamma transfer functions corresponding to the three visible clusters in the dendrogram are shown in Figure 11(b). The gamma transfer function in red was identified as statistically significant. It has a delay of approximately 2.5 s and a peak time of about 1.5 s.

Table 1 shows the number of significant clusters *C*_*S*_ identified for an optimal cluster division *C*_*N*_. The values shown correspond to the results from clustering recovered parameters of the gamma transfer function fit applied to EEG-NIRS data measured contralateral to the side of the median nerve stimulation.

## 4. Conclusion

Our mathematical model of neurovascular coupling is able to show the relationship between NIRS ΔHbO and EEG spectral envelopes based on gamma transfer function fits with clustered parameter sets. In case of simulated EEG-NIRS data, the correlations between the predicted ΔHbO and the simulated ΔHbO are approximately 1 for zero WGN. The recovered gamma transfer function parameters (see Figure 5) are identical to the original ones in the absence of WGN (AR = −*∞* dB). At higher noise amplitude ratios in WGN, the correlations drop almost linearly for all 5 NIRS datasets and we can still recover parameters of the gamma transfer function fits.

From the analysis of experimental EEG-NIRS data, we observed that multiple gamma transfer functions can significantly predict NIRS ΔHbO hemodynamic response from EEG spectral envelopes representing mu rhythm desynchronization events. By clustering the recovered parameters of the gamma functions including the estimated gains and offsets, we found statistically significant gamma transfer functions that can predict NIRS ΔHbO from EEG spectral envelopes. Those representative gamma transfer functions are characterized by different onset times and peak latencies in individual subjects (see Figures 9 and 11). These differences could be attributed to variability in the neural and hemodynamic response across brain regions including individual trials as suggested by previous studies [17, 19, 45].

This new method of analyzing the neurovascular coupling relationship has several potential advantages. It offers greater flexibility in modeling the relationship between EEG and NIRS data based on gamma transfer functions that can vary in their parameters. Using fixed transfer functions can introduce bias in modeling the cerebrovascular response and lead to inaccurate assessment of the onset times and latencies in neural activation [46, 47]. Also in fitting the gamma transfer functions, there are only five parameters to estimate. Hence, there is less potential for error in estimating them compared to models with a large number of parameters. Finally, we are using a data driven approach requiring minimal assumptions about the underlying biophysical process associated with neurovascular coupling. This makes the neurovascular coupling modeling problem more tractable and applicable in neuroimaging studies.

However, there may be some limitations in our approach to modeling the neurovascular coupling relationship. There is the potential for overfitting the data by allowing each gamma transfer function to have distinct parameters. We expect to overcome this problem by using the representative gamma transfer function derived from taking the average of the statistically significant parameter sets recovered from the model fitting of EEG-NIRS data measured from individual subjects. Due to the small number of human subjects, it is not possible to generalize about the nature of neurovascular coupling from the estimated parameters. However, the statistical testing was performed on an individual subject basis with a large number of feature vectors for each subject. This data was sufficient to allow us to develop this methodology. Nevertheless, acquiring EEG-NIRS data from a larger pool of subjects is necessary to validate neurovascular-coupling parameters that can be computed with this method.

Our method could be eventually extended to study the neurovascular coupling relationship in patients with neurodegenerative disorders. Several studies have reported that neurovascular coupling can be disrupted by brain disorders like Alzheimer's disease (AD), stroke, and epilepsy [48]. Certain brain disorders may cause changes in the chemical mediators of neurovascular coupling, which could lead to abnormal patterns of vasodilation and prevent removal of harmful byproducts of molecular metabolism. Ischemic stroke in the brain for instance can limit adequate flow of oxygenated blood to active neurons leading to vasoparalysis and impairing brain function [49]. We hope that we can apply our analysis method to detect abnormal neurovascular coupling in clinical neuroimaging studies using multimodal EEG-NIRS systems. Based on the data collected from patients, it will be possible to identify clustered gamma function parameters that could be significantly different from normal healthy populations.

## Figures and Tables

**Table 1:** Summary of the number of significant clusters *C*_*S*_ (*P* < 0.05) found for optimal cluster division. The number in parenthesis indicates the number of significant clusters as proportion.

Subject	Optimal *C*_*N*_, left stim.	*C*_*S*_, left stim.	Optimal *C*_*N*_, right stim.	*C*_*S*_, right stim.
1	7	4 0.57	5	2 0.4
2	2	2 (1)	3	1 0.33
3	5	3 0.6	3	2 0.67

## References

[1] Horwitz B., Friston K. J., Taylor J. G. (2000). Neural modeling and functional brain imaging: an overview. *Neural Networks*.

[2] Ferrari M., Quaresima V. (2012). A brief review on the history of human functional near-infrared spectroscopy (fNIRS) development and fields of application. *NeuroImage*.

[3] Debener S., Ullsperger M., Siegel M., Engel A. K. (2006). Single-trial EEG-fMRI reveals the dynamics of cognitive function. *Trends in Cognitive Sciences*.

[4] Martínez-Montes E., Valdés-Sosa P. A., Miwakeichi F., Goldman R. I., Cohen M. S. (2004). Concurrent EEG/fMRI analysis by multiway partial least squares. *NeuroImage*.

[5] Horovitz S. G., Rossion B., Skudlarski P., Gore J. C. (2004). Parametric design and correlational analyses help integrating fMRI and electrophysiological data during face processing. *NeuroImage*.

[6] Mullinger K., Debener S., Coxon R., Bowtell R. (2008). Effects of simultaneous EEG recording on MRI data quality at 1.5, 3 and 7 tesla. *International Journal of Psychophysiology*.

[7] Yamamoto T., Kato T. (2002). Paradoxical correlation between signal in functional magnetic resonance imaging and deoxygenated haemoglobin content in capillaries: a new theoretical explanation. *Physics in Medicine and Biology*.

[8] Rasmussen P., Dawson E. A., Nybo L., van Lieshout J. J., Secher N. H., Gjedde A. (2007). Capillary-oxygenation-level-dependent near-infrared spectrometry in frontal lobe of humans. *Journal of Cerebral Blood Flow & Metabolism*.

[9] Pfurtscheller G., Daly I., Bauernfeind G., Müller-Putz G. R. (2012). Coupling between intrinsic prefrontal Hbo2 and central EEG beta power oscillations in the resting brain. *PLoS ONE*.

[10] Herrmann M. J., Huter T., Plichta M. M. (2008). Enhancement of activity of the primary visual cortex during processing of emotional stimuli as measured with event-related functional near-infrared spectroscopy and event-related potentials. *Human Brain Mapping*.

[11] Koch S. P., Koendgen S., Bourayou R., Steinbrink J., Obrig H. (2008). Individual alpha-frequency correlates with amplitude of visual evoked potential and hemodynamic response. *NeuroImage*.

[12] Worsley K. J., Friston K. J. (1995). Analysis of fMRI time-series revisited—again. *NeuroImage*.

[13] Brookings T., Ortigue S., Grafton S., Carlson J. (2009). Using ICA and realistic BOLD models to obtain joint EEG/fMRI solutions to the problem of source localization. *NeuroImage*.

[14] Glover G. H. (1999). Deconvolution of impulse response in event-related BOLD fMRI. *NeuroImage*.

[15] Friston K. J., Holmes A. P., Worsley K. J., Poline J.-P., Frith C. D., Frackowiak R. S. J. (1994). Statistical parametric maps in functional imaging: a general linear approach. *Human Brain Mapping*.

[16] Cohen-Adad J., Chapuisat S., Doyon J. (2007). Activation detection in diffuse optical imaging by means of the general linear model. *Medical Image Analysis*.

[17] Handwerker D. A., Ollinger J. M., D'Esposito M. (2004). Variation of BOLD hemodynamic responses across subjects and brain regions and their effects on statistical analyses. *NeuroImage*.

[18] Boynton G. M., Engel S. A., Heeger D. J. (2012). Linear systems analysis of the fMRI signal. *NeuroImage*.

[19] Huppert T. J., Hoge R. D., Diamond S. G., Franceschini M. A., Boas D. A. (2006). A temporal comparison of BOLD, ASL, and NIRS hemodynamic responses to motor stimuli in adult humans. *NeuroImage*.

[20] Takeuchi M., Hori E., Takamoto K. (2009). Brain cortical mapping by simultaneous recording of functional near infrared spectroscopy and electroencephalograms from the whole brain during right median nerve stimulation. *Brain Topography*.

[21] Tanosaki M., Hoshi Y., Iguchi Y., Oikawa Y., Oda I., Oda M. (2001). Variation of temporal characteristics in human cerebral hemodynamic responses to electric median nerve stimulation: a near-infrared spectroscopic study. *Neuroscience Letters*.

[22] Giacometti P., Diamond S. G. (2013). Compliant head probe for positioning electroencephalography electrodes and near-infrared spectroscopy optodes. *Journal of Biomedical Optics*.

[23] Hoshi Y., Shimada M., Sato C., Iguchi Y. (2005). Reevaluation of near-infrared light propagation in the adult human head: implications for functional near-infrared spectroscopy. *Journal of Biomedical Optics*.

[24] Bell A. J., Sejnowski T. J. (1995). A non-linear information maximisation algorithm that performs blind separation. *Advances in Neural Information Processing Systems*.

[25] Jung T.-P., Makeig S., Westerfield M., Townsend J., Courchesne E., Sejnowski T. J. (2000). Removal of eye activity artifacts from visual event-related potentials in normal and clinical subjects. *Clinical Neurophysiology*.

[26] Mantini D., Perrucci M. G., Cugini S., Ferretti A., Romani G. L., del Gratta C. (2007). Complete artifact removal for EEG recorded during continuous fMRI using independent component analysis. *NeuroImage*.

[27] Hoffmann S., Falkenstein M. (2008). The correction of eye blink artefacts in the EEG: a comparison of two prominent methods. *PLoS ONE*.

[28] Flandrin P., Rilling G., Gonçalvés P. (2004). Empirical mode decomposition as a filter bank. *IEEE Signal Processing Letters*.

[29] Qian S., Chen D. (1999). Joint time-frequency analysis. *IEEE Signal Processing Magazine*.

[30] Addison P. S. (2005). Wavelet transforms and the ECG: a review. *Physiological Measurement*.

[31] Liang H., Bressler S. L., Desimone R., Fries P. (2005). Empirical mode decomposition: a method for analyzing neural data. *Neurocomputing*.

[32] Mehboob Z., Yin H. (2011). Information quantification of empirical mode decomposition and applications to field potentials. *International Journal of Neural Systems*.

[33] Talukdar T., Moore J. H., Diamond S. G. (2013). Continuous correction of differential path length factor in near-infrared spectroscopy. *Journal of Biomedical Optics*.

[34] Villringer A., Chance B. (1997). Non-invasive optical spectroscopy and imaging of human brain function. *Trends in Neurosciences*.

[35] Holman J. P., Gajda W. J. (1994). *Experimental Methods for Engineers*.

[36] Huang N. E., Shen S. S. (2005). *Hilbert-Huang Transform and Its Applications*.

[37] Boynton G. M., Engel S. A., Glover G. H., Heeger D. J. (1996). Linear systems analysis of functional magnetic resonance imaging in human v1. *The Journal of Neuroscience*.

[38] Franceschini M. A., Fantini S., Thompson J. H., Culver J. P., Boas D. A. (2003). Hemodynamic evoked response of the sensorimotor cortex measured noninvasively with near-infrared optical imaging. *Psychophysiology*.

[39] Lagarias J. C., Reeds J. A., Wright M. H., Wright P. E. (1998). Convergence properties of the Nelder—Mead simplex method in low dimensions. *SIAM Journal on Optimization*.

[40] Muthukumaraswamy S. D., Johnson B. W. (2004). Primary motor cortex activation during action observation revealed by wavelet analysis of the EEG. *Clinical Neurophysiology*.

[41] Huppert T. J., Diamond S. G., Franceschini M. A., Boas D. A. (2009). Homer: a review of time-series analysis methods for near-infrared spectroscopy of the brain. *Applied Optics*.

[42] Han J., Kamber M., Pei J. (2006). *Data Mining: Concepts and Techniques*.

[43] Ferreira L., Hitchcock D. B. (2009). A comparison of hierarchical methods for clustering functional data. *Communications in Statistics. Simulation and Computation*.

[44] Hogg R. V., Ledolter J. (1992). *Applied Statistics for Engineers and Physical Scientists*.

[45] Jasdzewski G., Strangman G., Wagner J., Kwong K. K., Poldrack R. A., Boas D. A. (2003). Differences in the hemodynamic response to event-related motor and visual paradigms as measured by near-infrared spectroscopy. *NeuroImage*.

[46] Bellgowan P. S. F., Saad Z. S., Bandettini P. A. (2003). Understanding neural system dynamics through task modulation and measurement of functional MRI amplitude, latency, and width. *Proceedings of the National Academy of Sciences of the United States of America*.

[47] Richter W., Somorjai R., Summers R. (2000). Motor area activity during mental rotation studied by time-resolved single-trial fMRI. *Journal of Cognitive Neuroscience*.

[48] Girouard H., Iadecola C. (2006). Neurovascular coupling in the normal brain and in hypertension, stroke, and Alzheimer disease. *Journal of Applied Physiology*.

[49] Iadecola C. (1998). Cerebral circulatory dysregulation in ischemia. *Cerebrovascular Diseases*.

